# The Taxonomic and Phylogenetic Affinities of *Bunopithecus sericus*, a Fossil Hylobatid from the Pleistocene of China

**DOI:** 10.1371/journal.pone.0131206

**Published:** 2015-07-08

**Authors:** Alejandra Ortiz, Varsha Pilbrow, Catalina I. Villamil, Jessica G. Korsgaard, Shara E. Bailey, Terry Harrison

**Affiliations:** 1 Center for the Study of Human Origins, Department of Anthropology, New York University, New York, New York, United States of America; 2 New York Consortium in Evolutionary Primatology (NYCEP), New York, New York, United States of America; 3 Department of Anatomy and Neuroscience, University of Melbourne, Melbourne, Victoria, Australia; Team 'Evo-Devo of Vertebrate Dentition', FRANCE

## Abstract

Fossil hylobatids are rare, but are known from late Miocene and Pleistocene sites throughout East Asia. The best-known fossil hylobatid from the Pleistocene of China is a left mandibular fragment with M_2-3_ (AMNH 18534), recovered from a pit deposit near the village of Yanjinggou in Wanzhou District, Chongqing Province. Matthew and Granger described this specimen in 1923 as a new genus and species, *Bunopithecus sericus*. Establishing the age of *Bunopithecus* has proved difficult because the Yanjinggou collection represents a mixed fauna of different ages, but it likely comes from early or middle Pleistocene deposits. Although the *Bunopithecus* specimen has featured prominently in discussions of hylobatid evolution and nomenclature, its systematic status has never been satisfactorily resolved. The present study reexamines the taxonomic and phylogenetic relationships of *Bunopithecus* by carrying out a detailed comparative morphometric study of its lower molars in relation to a large sample of modern hylobatids. Our results show that differences in M_2_ and M_3_ discriminate extant hylobatids fairly well, at least at the generic level, and that AMNH 18534 is not attributable to *Hylobates*, *Nomascus* or *Symphalangus*. Support for a close relationship between *Bunopithecus* and *Hoolock* is more equivocal. In most multivariate analyses, *Bunopithecus* presents a unique morphological pattern that falls outside the range of variation of any hylobatid taxon, although its distance from the cluster represented by extant hoolocks is relatively small. Our results support the generic distinction of *Bunopithecus*, which most likely represents an extinct crown hylobatid, and one that may possibly represent the sister taxon to *Hoolock*.

## Introduction

The fossil record documenting the evolutionary history of hylobatids (i.e., gibbons and siamangs) is extremely meager; the only definitive representatives of the family are known from localities in Asia dating to the late Miocene and Pleistocene. *Yuanmoupithecus* from the late Miocene (~7–8 Ma) of Yunnan, China [1] is considered a stem hylobatid based on the presence of a suite of dental specializations that are shared uniquely with extant members of the clade [2–3]. Pleistocene hylobatids are known from China, Vietnam, Thailand, Laos, Borneo, Sumatra and Java [3–26]. These are mostly isolated teeth, making their taxonomic and phylogenetic affinities difficult to ascertain, but they probably represent crown hylobatids and are likely attributable to extant genera [3, 22]. The earliest records of fossil hylobatids from the Quaternary of Asia come from cave sites in Guangxi in southern China with an estimated age of 2.2 Ma [26–27]. From the early Pleistocene onwards, and even into historic times, gibbons were widely distributed across southern China [6–7, 22, 28–30], whereas today they are restricted to Yunnan, Guangxi and Tibet in southwestern China and to the island of Hainan [31–34]. The best-known fossil hylobatid from the Pleistocene of China is a partial mandible from Yanjinggou in Chongqing Province (formerly part of Sichuan Province). The specimen was discovered by Walter Granger in 1920–1921 and was later described by Matthew and Granger [35] as a new genus and species, *Bunopithecus sericus*. Even though its phylogenetic affinities have never been satisfactorily resolved, *Bunopithecus* has featured prominently in discussions of hylobatid evolution and nomenclature.

**Fig 1:**
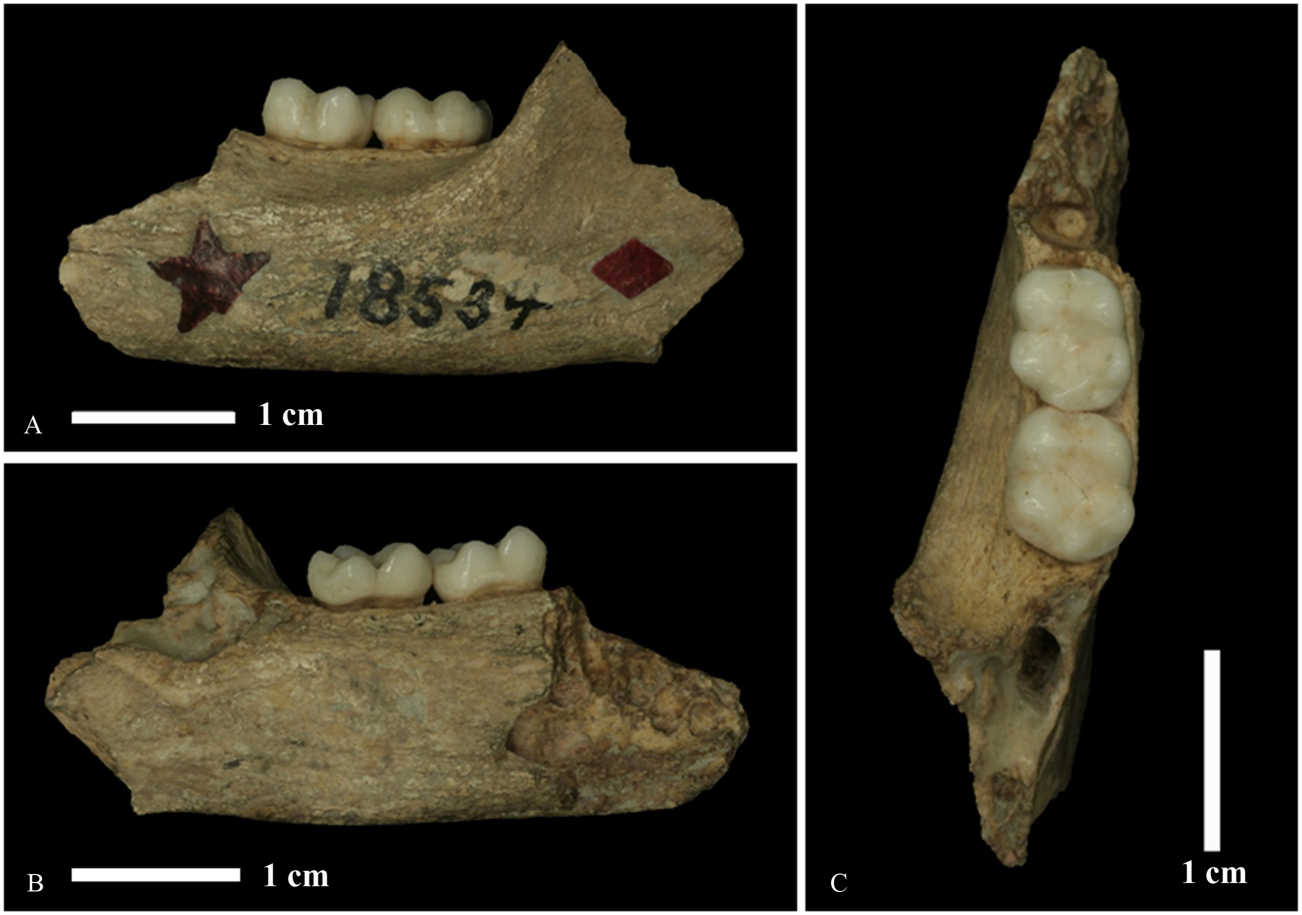
Photograph of the *Bunopithecus sericus* specimen (AMNH 18534) represented by a left mandibular fragment with M_2-3_. A) Lateral (buccal) view. B) Medial (lingual) view. C) Occlusal view (lingual to the right).

The *Bunopithecus sericus* type specimen (AMNH 18534) consists of a left mandibular fragment with M_2-3_ (Fig 1). The mandibular corpus is complete below M_2_ and M_3_ and the inferior margin extends anteriorly below the roots of P_4_ and M_1_. Posterior to M_3_, the anterior portion of the ramus is also preserved. The two molars are well-preserved and only lightly worn. The specimen was recovered from the pits and fissures near the village of Yanjinggou in Wanzhou District, Chongqing (Yen-ching-kao, Wan-hsien, Szechuan of [35]). Although Yanjinggou is one of the most famous and productive Quaternary fossil vertebrate sites in China, establishing the age of the fauna has proved difficult. Matthew and Granger [35] provisionally attributed the Yanjinggou fauna to the late Pliocene, but subsequent studies indicated an early to late Pleistocene age [36–39]. The major impediment to ascertaining the age of Granger’s collection is that it probably represents a mixed fauna of different ages [40]. Recent renewed field work at Yanjinggou has helped to clarify the ages of the faunas, and it appears most likely that the *Bunopithecus* specimen came from early or middle Pleistocene deposits [40].

Matthew and Granger [35] noted similarities between the Yanjinggou specimen and extant gibbons, but opted to name a new genus and species for the fossil due to several distinctive features of the molars (i.e., greater crown width and larger hypoconulid size). Colbert and Hooijer [38], using a larger comparative sample, argued that the purported distinctive dental features of *B*. *sericus* are found among extant gibbons. They concluded that the generic distinction was unwarranted, and preferred to recognize the fossil as *Hylobates* (*Bunopithecus*) *sericus*. Subsequently, Frisch [41] and Groves [29] inferred that *B*. *sericus* was most closely related to hoolock gibbons. With the recognition that hoolock gibbons should be considered a distinct subgenus/genus [42], the apparent close relationship between *Bunopithecus sericus* and extant hoolock gibbons became central to deciding on an available genus-group name [43]. If the fossil species is included in the same genus, then *Bunopithecus* Matthew and Granger, 1923 becomes the oldest available name for hoolock gibbons (and one of the rare examples of a fossil type species for an extant genus). For the next two decades the subgenus/genus name *Bunopithecus* was widely employed as the valid name for the hoolock gibbons. However, further investigations cast doubt on the dental similarities between extant hoolocks and *Bunopithecus*, and led to a rethinking of their taxonomic association. Gu [7] suggested, without offering much in the way of supporting morphological evidence, that *B*. *sericus* most closely resembles *Nomascus concolor*. In their review of gibbon evolution, Jablonski and Chaplin [22] followed Gu [7] in referring *Bunopithecus sericus* to *Nomascus* (even though *Bunopithecus* Walter and Granger, 1923 has priority over *Nomascus* Miller, 1933). Most recently, Mootnick and Groves [44] pointed out that the dental characters of *B*. *sericus* fall outside the range of variation of extant gibbons and hypothesized that *Bunopithecus* probably represents an extinct genus, without a close evolutionary relationship to hoolock gibbons. In light of this, Mootnick and Groves [44] proposed a new genus name, *Hoolock*, for the extant hoolock gibbons, and excluded *Bunopithecus* from synonymy.

Currently, the affinities of *Bunopithecus sericus* remain unsettled. The main problems hampering previous interpretations of its taxonomic status are that the fossil is represented by a single fragmentary type specimen, that only small samples of modern gibbons have been used in comparisons, that the cheek teeth of extant hylobatids are notoriously difficult to discriminate (except by differences in size), and that most assessments have been based on just a few linear measurements and qualitative traits [35, 38, 41, 43, 45–46]. The present study aims to clarify the taxonomic and phylogenetic relationships of *Bunopithecus* by carrying out a detailed comparative morphometric study of its lower molars in relation to a large sample of modern hylobatids.

Extant hylobatids are currently included in four genera–*Hylobates*, *Hoolock*, *Symphalangus* and *Nomascus*. The phylogenetic relationships between the extant genera have not been adequately resolved [47–61], and the lack of consensus probably stems from the rapid radiation of the crown taxa and the confounding influences of species introgression [58, 61]. Nevertheless, the majority of studies provide support for the following set of relationships: (*Nomascus* (*Symphalangus* (*Hoolock*, *Hylobates*))) [47–56, 60]. Molecular clock estimates indicate a date of ~19 Ma (with a range of ~16.3–21.8 Ma) for the divergence of the hylobatids from the other hominoids and ~8.4 Ma (with a range of 10.5–5.2 Ma) for the divergence of the extant genera [3, 47–56, 60, 62–65]. Given this inferred chronology, *Bunopithecus* postdates the differentiation of the extant generic lineages, which increases the likelihood that the fossil belongs to a member of the crown clade, but it does not entirely rule out the possibility that it represents a late-surviving stem hylobatid. The enduring question then is whether *Bunopithecus* is distinct enough to be retained as a valid genus (either as a stem hylobatid or as a distinctive crown clade) or whether it should be subsumed into one of the four currently recognized genera of extant hylobatids (and if so, which one?). If *Bunopithecus* is deemed to belong to one of the extant hylobatid genera, then priority of the name *Bunopithecus* over both *Hoolock* and *Nomascus* becomes a critical nomenclatural issue that will need to be considered. In addition to clarifying the taxonomic status of *Bunopithecus*, the results of our study have implications for understanding hylobatid biogeography and evolutionary relationships.

## Materials and Methods

### Samples

The present study examines the M_2_ and M_3_ of the *B*. *sericus* fossil specimen and makes comparisons with those of extant hylobatids. Access to the original *B*. *sericus* type specimen (AMNH 18534) was given to AO by the Division of Vertebrate Paleontology at the American Museum of Natural History (AMNH), New York, United States of America. Permission to study the fossil was notified by Ms. Judith Galkin from the Division of Vertebrate Paleontology, AMNH via email on 05/15/13. A total of 289 molar teeth represented by 172 extant individuals from *Hylobates*, *Hoolock*, *Nomascus* and *Symphalangus* were included in the comparative sample (Table 1). It should be noted that the sample size per molar type varies due to missing teeth and differential preservation and wear. Metrical data on extant hylobatids were collected from the following museums: AMNH; National Museum of Natural History (NMNH), Washington D.C.; Field Museum of Natural History (FMNH), Chicago; Museum of Comparative Zoology (MCZ), Cambridge; Natural History Museum (BMNH), London; Zoologisches Museum (ZMB), Berlin; Anthropologische und Zoologische Staassammlung (ZSM), Munich; Anthropologisches Institüt und Museum der Universität Zurich-Irchel (AS/Z), Zurich; and Muséum National d’Histoire Naturelle (MNHN), Paris. Only data from individuals of known provenance were collected. Provenance information was obtained from museum records and the nomenclature was updated to reflect the currently accepted taxonomy [32, 34]. Because molar size is not sexually dimorphic in extant hylobatids [46], sex was not included as a variable in this study. We attempted, however, to maintain an equal representation of male and female individuals. Information on dental wear was collected using Pilbrow’s [66] three-stage system and specimens with heavily worn teeth were excluded from analyses.

**Table 1:** Fossil and recent comparative sample of hylobatid lower molars used in this study.

Taxon	Number of individuals	Number of molars	M_2_	M_3_
***Bunopithecus sericus***	1	2	1	1
***Hoolock hoolock***	20	32	19	13
***Nomascus concolor***	17	34	17	17
***Symphalangus syndactylus***	30	50	28	22
***Hylobates*** *	105	173	100	73

*This sample includes representatives of the following species: *H*. *agilis* (M_2_ = 32, M_3_ = 20), *H*. *albibarbis* (M_2_ = 9, M_3_ = 6), *H*. *klossi* (M_2_ = 10, M_3_ = 8), *H*. *lar* (M_2_ = 12, M_3_ = 11), *H*. *moloch* (M_2_ = 9, M_3_ = 8), *H*. *muelleri* (M_2_ = 22, M_3_ = 16), *H*. *pileatus* (M_2_ = 4, M_3_ = 4), and *Hylobates* sp. (M_2_ = 2, M_3_ = 0).

### Data Collection Procedures

High-resolution images of the occlusal surface of the M_2_ and M_3_ of *B*. *sericus* and those of the comparative samples were taken with either a Canon Digital Rebel XT camera with 18–55mm lens (AO) or a Minolta X-700 camera outfitted with a Sigma 50mm F2.8 macro lens (VP). As described elsewhere [66–67], teeth were positioned so that the cervical border was perpendicular to the optical axis of the camera. A millimeter scale was included in each image, placed at the same horizontal plane as the cusp apices, and both the scale and the camera were leveled using standard bubble devices. The smallest aperture possible was used to maximize depth of field. Because only the left mandibular corpus is preserved in *B*. *sericus*, comparative data were collected on left molars when available. In the case of missing or damaged teeth, the right antimere was used and then digitally mirror-imaged to correspond to the left side. Data were collected separately for each molar type.

Digital images were imported into SigmaScan Pro (Systat Software Inc.) imaging software in order to collect mesiodistal and buccolingual dimensions, the angle between cusps (i.e., cusp position), and cusp and crown base areas. All measurements used in our analysis have proven useful for differentiating between fossil and living hominins [68–71] and great apes [66, 72–74]. Importantly, previous studies have shown that intra and inter-observer errors in tooth crown orientation and measurement are relatively low (0.5–3.0%) and not statistically significant [66, 75].

**Fig 2:**
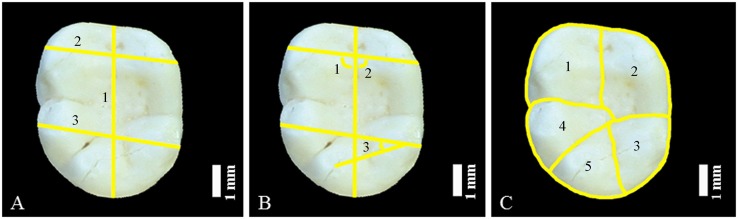
Hylobatid left lower molar showing dental variables taken. A) Linear dimensions: 1) mesiodistal length, 2) buccolingual width at mesial cusps, and 3) buccolingual width at distal cusps. B) Angles: 1) position of mesiobuccal cusp, 2) position of mesiolingual cusp, and 3) position of hypoconulid. C) Absolute cusp areas: 1) protoconid, 2) metaconid, 3) entoconid, 4) hypoconid, and 5) hypoconulid.

As defined by Pilbrow [66], the mesiodistal length (MDLENGTH) was measured along the longitudinal developmental groove (Fig 2). Buccolingual breadths were taken at the tips of the mesial (BLMES) and distal (BLDIS) cusps. The positions of the protoconid (ANBCUSP) and metaconid (ANLCUSP) were collected as the angles formed between the lines connecting the tips of these cusps to the buccal and lingual sides of the longitudinal groove, respectively. Similarly, the position of the hypoconulid (ANHYCLD) was calculated as the angle formed by the apices of the entoconid, hypoconid and hypoconulid. Individual areas for the protoconid (ABSAPROTO), metaconid (ABSAMETA), entoconid (ABSAENTO), hypoconid (ABSAHYPCD) and hypoconulid (ABSAHYPCLD) were measured by tracing the outline of the crown and the main fissures dividing the cusps. Wood and Engleman’s [76] protocol was followed when minor corrections for interproximal wear were necessary, as well as for estimating the individual areas of the five main cusps in cases where additional cusps (e.g., C6, C7) or marginal tubercles were present. The total crown area (OCCLAREA) was calculated by summing the individual cusp areas. Similarly, the sum of the areas of the protoconid and metaconid and the areas of the hypoconid, hypoconulid and entoconid gave the total area of the trigonid (ABSATRIGD) and talonid (ABSATALD), respectively. Relative cusp areas (RELAPROTO, RELAMETA, RELAENTO, RELAHYPCD and RELAHYPCLD) were determined by dividing the individual cusp areas by the total crown area. The same protocol was used for calculating the relative trigonid (RELATRIGD) and talonid (RELATALD) areas. A total of 21 variables were analyzed for each molar.

### Statistical Analyses

Univariate and multivariate statistical analyses were performed on PAST [77]. Unless otherwise noted, differences were considered significant at α = 0.05. Because sample sizes differ between taxa and the data are non-parametric, Kruskal-Wallis tests were performed for each variable to determine whether sample medians were significantly different from each other. Mann-Whitney *U* tests were performed on each variable to determine where such differences lay, with *p*-values subjected to a Bonferroni correction. Z-tests were also performed on the logged data to determine whether *B*. *sericus*, for which a single data point is available for each variable, can be excluded from each extant group. To determine group differences and the identity of *B*. *sericus* using all available data, discriminant function analyses (DFAs) were performed using both absolute values that contain information about size and relative values that contain information primarily on shape. Linear measurements BLMES and BLDIS were standardized relative to MDlength, whereas measurements of cusp areas were standardized as a proportion of the total occlusal area. Either absolute or relative cuspal occlusal area values were used, with the total occlusal area itself excluded from all DFAs to avoid excessive weighting of tooth area in the results. Angle measurements were converted to radians for all DFAs to normalize their distribution and reduce their range of variation. DFAs were performed for M_2_ and M_3_ separately and combined. Accuracy of the DFAs was quantified by determining the percent of individuals correctly classified, with and without jackknifing. Individuals with missing data were not included in analyses.

## Results

Descriptive statistics for the 21 variables of M_2_ and M_3_ for both *B*. *sericus* and extant hylobatids are presented in S1 and S2 Tables, respectively. Results of the Kruskal-Wallis statistical analysis are presented in Table 2 and details of the pair-wise Mann-Whitney *U* tests are provided in S3 Table. Our results show that size has a greater discriminatory power than shape in both M_2_ and M_3_, with all linear dimensions and absolute areas showing significant differences among sample medians (Table 2). With few exceptions (e.g., the position and relative size of the hypoconulid), shape variables such as cusp angles and relative areas do not appear useful for differentiating among hylobatid taxa. Previous studies have argued that due to the existence of only minor differences in the dental morphology between genera and the high degree of intra-specific variability, dental characters are not particularly informative for hylobatid systematics [22, 41, 45–46]. However, the results of the Mann-Whitney *U* tests performed on the samples included in this study reveal the potential of several dental variables to differentiate among groups, at least at the generic level. For M_2_, the following variables are significantly different between sample medians in all four extant genera: MDlength, ABSAHYPCD, ABSAHYPCLD (except for *Hoolock* vs. *Nomascus*), ABSATRIGD, ABSATALD and OCCLAREA (S3 Table). Similarly, significant differences in all pair-wise comparisons were found for each of the following M_3_ variables: MDlength (except for *Hylobates* vs. *Nomascus*), ABSAHYPCD (except for *Hoolock* vs. *Nomascus*), ABSATRIGD, ABSATALD (except for *Hylobates* vs. *Nomascus*) and OCCLAREA (S3 Table). In general, among extant hylobatids, *Symphalangus* shows the largest mean values for each of the absolute cusp areas, and concomitantly the largest mean values for the trigonid, talonid and total occlusal base. Molar size is second largest in *Hoolock*, followed by *Nomascus* and finally *Hylobates*, which shows the smallest absolute size with regards to the variables examined (S1 and S2 Tables).

**Table 2:** Kruskal-Wallis test for differences between sample medians.

Features	M_2_		M_3_	
	H(χ^2^)	*p*-value*	H(χ^2^)	*p*-value*
MDLENGTH	39.54	< **0.001**	70.96	< **0.001**
BLMES	11.96	< **0.01**	31.14	< **0.001**
BLDIS	20.14	< **0.001**	36.15	< **0.001**
ANBCUSP	-16.33	1	3.48	0.323
ANLCUSP	-12.12	1	3.628	0.305
ANHYCLD	0.6703	0.88	9.49	< **0.05**
ABSAPROTO	10.46	< **0.01**	53.86	< **0.001**
ABSAHYPCD	54.97	< **0.001**	62.37	< **0.001**
ABSAHYPCLD	54.14	< **0.001**	47.83	< **0.001**
ABSAMETA	44.22	< **0.001**	58.18	< **0.001**
ABSAENTO	35.67	< **0.001**	32.35	< **0.001**
OCCLAREA	74.61	< **0.001**	76.61	< **0.001**
ABSATRIGD	57.81	< **0.001**	64.7	< **0.001**
ABSATALD	70.24	< **0.001**	71.58	< **0.001**
RELAPROTO	7.645	0.054	4.793	0.188
RELAHYPCD	2.643	0.45	6.204	0.102
RELAHYPCLD	21.12	< **0.001**	20.32	< **0.001**
RELAMETA	6.798	0.078	7.392	0.06
RELAENTO	-1.695	1	7.296	0.063
RELATRIGD	13.77	< **0.01**	3.867	0.276
RELATALD	12.45	< **0.01**	19.72	< **0.001**

* Significant values bolded.

S1 and S2 Tables also provide the values for each variable for the *B*. *sericus* specimen. Comparisons with the samples of extant genera show that most absolute values for *B*. *sericus* approximate the mean values of *Hoolock* more closely than those of other taxa, although there is considerable overlap. Confidence intervals at the 95% confidence level (± 2SD) suggest that the M_2_ of *B*. *sericus* and *Symphalangus* are significantly different in MDlength and ABSAHYPCD. Similarly, there is a statistically significant difference between the M_2_ of *B*. *sericus* and *Nomascus* in BLMES, BLDIS and ANLCUSP, and between *B*. *sericus* and *Hylobates* in ABSATRIGD and OCCLAREA. Data for the M_3_ at the 95% confidence level of the absolute measurements and cusp position variables indicate that *B*. *sericus* significantly differs from *Nomascus* in BLMES, ANBCUSP and ANLCUSP and from *Hylobates* in MDlength and ABSAHYPCD. It differs significantly from both *Nomascus* and *Hylobates* in ABSAMETA, ABSATRIGD, ABSATALD and OCCLAREA. Finally, the ABSAHYPCLD of *B*. *sericus* M_3_ is significantly different from that of *Hoolock* and *Hylobates*.

The results of the Z-tests are summarized in Table 3. As noted above, these tests were conducted on each variable to determine whether *B*. *sericus* can be excluded from any of the extant hylobatid genera. Contrary to early claims by Matthew and Granger [35] using a small comparative sample, *B*. *sericus* has significantly narrower tooth crowns across the mesial cusps (BLMES) in both M_2_ and M_3_ than the four extant hylobatid genera (see also S1 and S2 Tables). In general, with the exception of ANBCUSP, all linear measurements, absolute areas and cusp angles in M_2_ indicate that *B*. *sericus* does not belong to *Symphalangus*. The exclusion of *B*. *sericus* as a member of *Symphalangus* based on information contained in M_3_ is, however, slightly less robust. Comparisons with the other three extant groups are also more ambiguous. In addition to BLMES, *B*. *sericus* significantly differs from extant gibbons in having a smaller relative hypoconid size (RELAHYPCD) in M_2_ and, except for the M_2_ of extant hoolocks, a smaller ANLCUSP in both M_2_ and M_3_. It is also excluded from *Nomascus* by BLDIS in both molars and by ANHYCLD in M_2_. The fossil significantly differs from *Hoolock* also in ANHYCLD for M_2_, and absolute (ABSAENTO) and relative (RELAENTO) entoconid size for M_3_, and from *Hylobates* in RELAMETA and RELATALD for M_2_, and RELAPROTO for M_3_. When information based on confidence intervals and Z-tests is contrasted for more robust assessments, significant values in agreement between univariate analyses demonstrate that *B*. *sericus* can be excluded from *Nomascus* by BLMES and ANLCUSP in both molars, and by BLDIS in M_3_. We also found that *B*. *sericus* does not align with *Symphalangus* in MDlength and ABSAHYPCD for M_2_.

**Table 3:** Significance of between-group comparisons based on Z-tests.

Features	*B*. *sericus* vs. *Hoolock*		*B*. *sericus* vs. *Hylobates*		*B*. *sericus* vs. *Nomascus*		*B*. *sericus* vs. *Symphalangus*	
	M_2_	M_3_	M_2_	M_3_	M_2_	M_3_	M_2_	M_3_
MDLENGTH	N.S.	N.S.	N.S.	N.S.	N.S.	N.S.	**	**
BLMES	**	**	**	**	**	**	**	**
BLDIS	N.S.	N.S.	N.S.	N.S.	**	**	**	**
ANBCUSP	N.S.	N.S.	N.S.	N.S.	N.S.	N.S.	N.S.	N.S.
ANLCUSP	N.S.	**	**	**	**	**	**	**
ANHYCLD	**	N.S.	N.S.	N.S.	**	N.S.	**	N.S.
ABSAPROTO	N.S.	N.S.	N.S.	N.S.	N.S.	N.S.	**	**
ABSAHYPCD	N.S.	N.S.	N.S.	N.S.	N.S.	N.S.	**	N.S.
ABSAHYPCLD	N.S.	N.S.	N.S.	N.S.	N.S.	N.S.	**	N.S.
ABSAMETA	N.S.	N.S.	N.S.	N.S.	N.S.	N.S.	**	**
ABSAENTO	N.S.	*	N.S.	N.S.	N.S.	N.S.	**	N.S.
OCCLAREA	N.S.	N.S.	N.S.	N.S.	N.S.	N.S.	**	**
ABSATRIGD	N.S.	N.S.	N.S.	N.S.	N.S.	N.S.	**	**
ABSATALD	N.S.	N.S.	N.S.	N.S.	N.S.	N.S.	**	**
RELAPROTO	N.S.	N.S.	N.S.	*	N.S.	N.S.	N.S.	N.S.
RELAHYPCD	*	N.S.	**	N.S.	*	N.S.	**	N.S.
RELAHYPCLD	N.S.	N.S.	N.S.	N.S.	N.S.	N.S.	**	N.S.
RELAMETA	N.S.	N.S.	**	N.S.	N.S.	N.S.	*	N.S.
RELAENTO	N.S.	*	N.S.	N.S.	N.S.	N.S.	N.S.	N.S.
RELATRIGD	N.S.	N.S.	N.S.	N.S.	N.S.	N.S.	*	N.S.
RELATALD	N.S.	N.S.	**	N.S.	N.S.	N.S.	N.S.	N.S.

* Significant at p<0.05;

**significant at p<0.01;

N.S.: non-significant.

**Fig 3:**
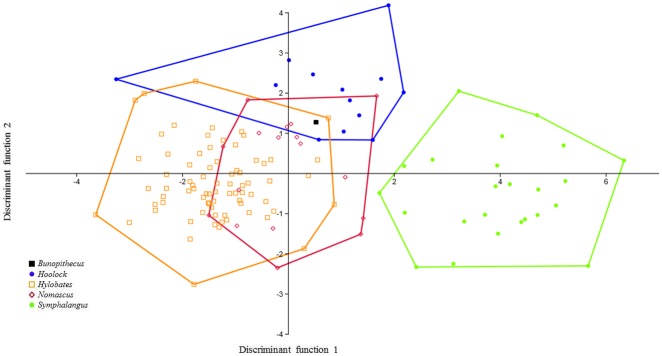
Plot of the first two discriminant functions (DF1 and DF2) of the M_2_ analysis using linear measurements, cusp angles and absolute areas. Eigenvalues: 4.19 (DF1) and 0.52 (DF2); variance: 84.44% (DF1) and 10.41% (DF2).

**Fig 4:**
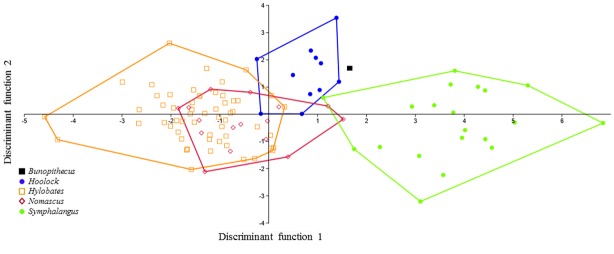
Plot of the first two discriminant functions (DF1 and DF2) of the M_3_ analysis using linear measurements, cusp angles and absolute areas. Eigenvalues: 40.22 (DF1) and 0.31 (DF2); variance: 87.07% (DF1) and 6.80% (DF2).

Figs 3 and 4 illustrate the plots of the first two discriminant functions for individual molars showing the relative placement of *B*. *sericus* using linear measurements, cusp angles and absolute areas. S4 Table provides the average Mahalanobis distances between hylobatid taxa for each analysis. For M_2_, the first function is responsible for 84.4% of the variance, while the following two functions explain 10.4% and 4.1%, respectively. Similar values were obtained for M_3_, as the first three functions account for the 87.1%, 6.8% and 4.1% of the variance, respectively. The DFAs performed on M_2_ and M_3_ independently reveal that the greatest degree of overlap among hylobatids occurs between *Nomascus* and *Hylobates*. As expected, given their larger dentitions, siamangs are the most distinctive of the hylobatids. As illustrated in Fig 3, the M_2_ of *B*. *sericus* falls within the range of overlap among *Nomascus*, *Hoolock* and *Hylobates*, but when all axes are considered, the average pair-wise Mahalanobis comparisons indicate a closest distance to hoolock gibbons (S4 Table). In contrast, data for M_3_ for the first two discriminant functions show that *B*. *sericus* does not cluster with any extant group, although distances between *B*. *sericus* and the cluster represented by hoolock gibbons are relatively small (Fig 4). Although *Bunopithecus*' average Mahalanobis distance is smaller to *Hylobates* (1.3104) than to *Hoolock* (1.3159), the average Mahalanobis distance within *Hylobates* is much smaller (1.2086) than within *Hoolock* (1.3828), which places *B*. *sericus* well within the possible spread of *Hoolock* but not within the spread of *Hylobates* (S4 Table). The likelihood of individuals being accurately classified ranges between 80.15% (not jackknifed) and 71.76% (jackknifed) for M_2_ and between 79.05% (not jackknifed) and 62.86% (jackknifed) for M_3_.

**Fig 5:**
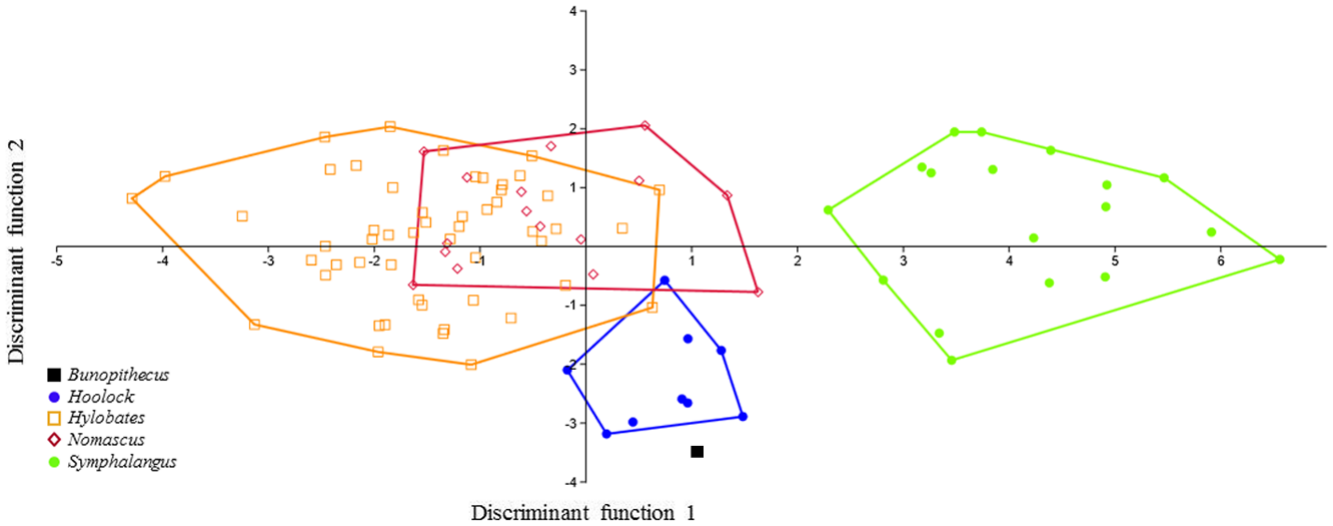
Plot of the first two discriminant functions (DF1 and DF2) in the analysis of size variables, M_2_ and M_3_ combined. Eigenvalues: 4.79 (DF1) and 0.73 (DF2); variance: 74.32% (DF1) and 11.41% (DF2).

When data for M_2_ and M_3_ are combined, the results of the DFA using the same set of variables (i.e., linear measurements, cusp angles and absolute areas) are slightly more robust with respect to grouping patterns among extant hylobatids. Separation among clusters and the relative position of *B*. *sericus* on the scatter plots of the first two discriminate functions are shown in Fig 5. The first function accounts for 74.32% of the variance, with ABSATRIGD and ABSATALD in both molars contributing the most to this axis. The second and third axes, on the other hand, encompass 11.41% and 9.46% of the variance, respectively. As illustrated in Fig 5, with the exception of the overlap between *Nomascus* and *Hylobates*, each genus is distinct. Again, *B*. *sericus* falls outside the range of variation observed among extant gibbons. However, the distance between *B*. *sericus* and the cluster represented by extant hoolock gibbons is quite small (see also S4 Table). The likelihood of individuals being correctly classified ranges between 90.63% (not jackknifed) and 62.50% (jackknifed), which is lower than for either M_2_ or M_3_ alone.

**Fig 6:**
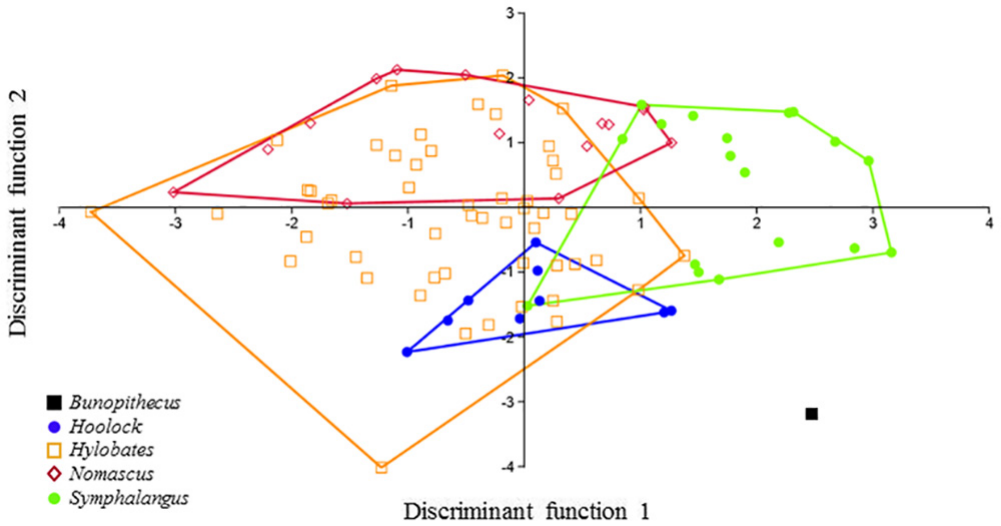
Plot of the first two discriminant functions (DF1 and DF2) in the analysis of shape variables, M_2_ and M_3_ combined. Eigenvalues: 0.95 (DF1) and 0.62 (DF2); variance: 42.88% (DF1) and 27.96% (DF2).

Finally, a DFA of M_2_ and M_3_ data combined was performed using exclusively shape variables, with absolute size components removed (Fig 6). In this analysis, the first function explains 42.88% of the variance, while the following two account for 27.96% and 19.36%, respectively. In agreement with results based on Kruskal-Wallis and Mann-Whitney *U* tests, this analysis demonstrates that shape variables alone have less discriminatory power than when size is considered, as shape variables alone result in no discernible differences between groups. Based on shape variables alone, *B*. *sericus* falls outside the range of variation of extant hylobatids, but closest to *Hoolock* (see also S4 Table). The likelihood of the classification of individuals into their correct group is relatively lower (75% not jackknifed and 40.63% jackknifed).

## Discussion

Our results show that differences in M_2_ and M_3_ discriminate modern hylobatids fairly well, at least at the generic level, and that these differences have the potential to contribute to the taxonomic identification of fossil members of the clade. This is noteworthy given that most previously recognized distinctions between hylobatid genera relate to soft tissue features (e.g., pelage and hair coloration patterns, ear size, nose shape, and hand and feet color) and karyology, although some skeletal differences in cranial size and shape, anatomy of the baculum, and number of thoracic vertebrae have also been reported [29, 78–80]. Informative dental features include MDlength, ABSATRIGD, ABSATALD and OCCLAREA, and to a lesser extent, the absolute area of each lower molar cusp. Since M_3_ exhibits a greater degree of intra-specific variability [81], M_2_ has a greater discriminatory power than that of the M_3_. Moreover, variables that contain size and shape data, rather than those containing information about shape alone, are considerably more useful for identifying taxonomic differences in extant hylobatids. In general, *Symphalangus* has the largest teeth, followed in decreasing order by *Hoolock*, then *Nomascus* and finally *Hylobates*. As shown by our study, the linear dimensions of *B*. *sericus* teeth overlap with those observed among all four extant hylobatid genera, although they fall closest to the mean values for *Hoolock*. However, it is necessary to be cautious about using overall dental size or size-based dental features in taxonomic assessments since there has been a general trend among many Asian mammals, including primates, to undergo dental size reduction during the course of the Pleistocene [9, 82–83]. As a consequence, shared morphological specializations are a much better guide to taxonomic affinities than dental size.

The results of our study show that *B*. *sericus* is unlikely to belong to *Hylobates*, *Nomascus* or *Symphalangus*. In fact, most of our univariate and multivariate analyses reveal that *B*. *sericus* exhibits the greatest differences with extant *Nomascus* and *Symphalangus*, especially when size variables are considered. In addition, as noted by Frisch [41], *Nomascus* is the only genus with a relatively high frequency (61.7%) of buccal cingula on the lower molars, a feature that is lacking in *B*. *sericus*. The presence of a lingual cingulum is also common on *Nomascus* upper molars and traces are occasionally observed in *Hylobates*. In contrast, hoolocks appear to have lost the cingulum on both upper and lower molars. Frisch [41] also noted that *Symphalangus* exhibits the least reduced lower third molars, with *Hoolock* and *Nomascus* occupying an intermediate position. Although highly variable, the metaconid tends to be distal to the protoconid and the hypoconulid is placed in the midline (or slightly buccal to the midline) in *Nomascus* and *Hoolock* molars, while in *Hylobates* the metaconid and protoconid are more transversely aligned and the hypoconulid is more lingually positioned [41, 46].

Support for a close relationship between *B*. *sericus* and modern hoolocks is more equivocal. Taxon means and confidence intervals for each variable, as well as DFA and Z-test results, suggest that although *B*. *sericus* is most similar to *Hoolock* relative to other hylobatid genera, it remains dentally distinct. In most multivariate analyses, *B*. *sericus* presents a unique morphological pattern that falls outside the range of variation of any hylobatid taxon, although its distance from the cluster represented by extant hoolocks is relatively small. Our findings support the conclusions reached by Mootnick and Groves [44] that *Bunopithecus* should be considered a distinct genus. Given the morphological and metrical evidence, it seems likely that *Bunopithecus* represents an extinct crown hylobatid, one that may possibly represent the sister taxon to *Hoolock*. However, since the *Hoolock* sample used in this study is relatively small (n = 20 individuals), and may not adequately encompass the full extent of the intra-generic variation, we cannot entirely rule out the possibility that *B*. *sericus* merely represents an early hoolock gibbon that should be included in the genus *Hoolock*. At present, given the results of our study, we prefer to recognize *Bunopithecus* as a separate genus.

**Fig 7:**
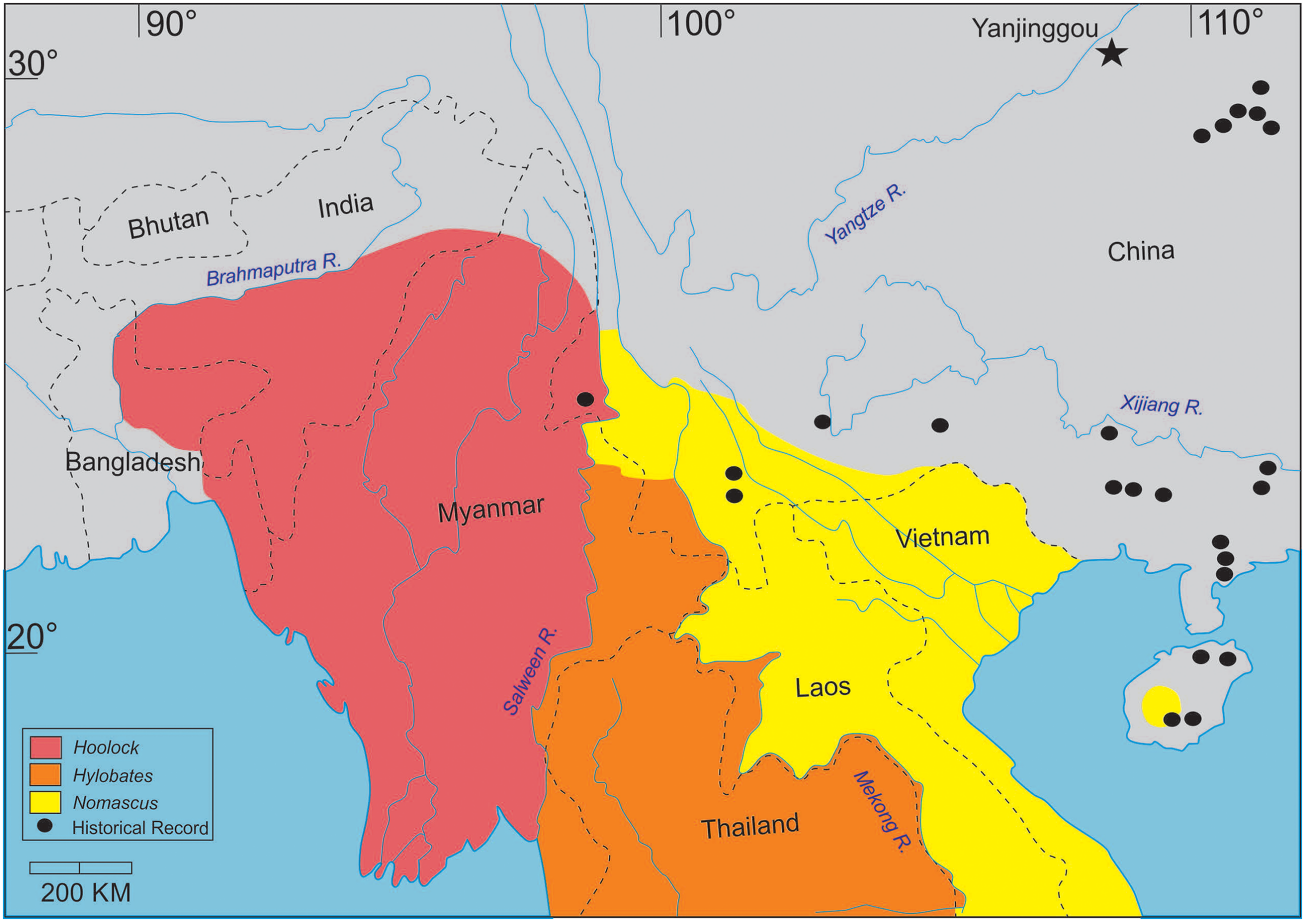
Map of East and Southeast Asia showing the historical and present distribution of gibbons (*Hoolock*, *Hylobates* and *Nomascus*). The black star indicates the location of the village of Yanjinggou (Wanzhou District, Chongqing Province, China), where *Bunopithecus sericus* was found. Adapted from Gu [7], Gao et al. [30] and Geissmann [80].

This opens up the intriguing possibility that an extinct gibbon taxon, in the form of *Bunopithecus*, may have occupied parts of China to the north and east of the current geographic distribution of extant gibbons during the Pleistocene-Holocene, and may have even survived into historic times (Fig 7). Evidence from historical records shows that gibbons in China were much more widely distributed in the recent past than they are today, extending as far north as the Yellow River and eastwards as far as Zhejiang Province [7, 28, 30, 80]. Records of hylobatids south of the Xijiang River are almost certainly attributable to *Nomascus*, and these serve to fill the present-day geographic divide between the disjunct distribution of *Nomascus* in western China, Vietnam and Laos and isolated populations on Hainan (Fig 7). In addition, teeth of fossil gibbons from cave sites in Guangxi Zhuang Autonomous Region are consistent in size with those of *Nomascus* and, like their modern counterparts, they retain a high incidence of cingula on the upper and lower molars [7]. These lines of evidence indicate that *Nomascus* occupied much of southern China from at least the Early Pleistocene onwards. The taxonomic identity of recently extirpated gibbons to the north of the Xijiang River is much harder to establish. Geissmann [80] has suggested that paintings of gibbons that were living in Hunan and Hubei Provinces in central China during the eleventh century are strikingly similar to *Hoolock*. This could potentially extend the geographic range of the genus eastwards more than 1,200 km beyond its present-day distribution. However, an alternative interpretation is conceivable. The historic records of gibbons from central China south of the Yangtze River may refer to an extinct genus of hylobatid (Fig 7). Since these occurrences are in the same general region as Yanjinggou, it is plausible that the extinct genus was represented during the Pleistocene by *Bunopithecus sericus*. In this case, the apparent similarities to *Hoolock* in the historical depictions of gibbons from central China would not be unexpected given that the results of our study indicate that *Bunopithecus* is likely to be the sister taxon of *Hoolock*. It may well be that the mandibular fragment from the Pleistocene of Yanjinggou represents an extinct hylobatid genus, *Bunopithecus*, which was once widely distributed across central and eastern China before becoming extinct in historic times.

## Supporting Information

S1 Table Descriptive statistics for M_2_ size and shape variables in *B*. *sericus* and extant hylobatid genera. (PDF)

S2 Table Descriptive statistics for M_3_ size and shape variables in *B*. *sericus* and extant hylobatid genera. (PDF)

S3 Table Extant hylobatid variance analysis. Left: Mann-Whitney *U* test values; Right: Bonferroni adjusted *p*-values with significant values bolded.
(PDF)

S4 Table Average pair-wise Mahalanobis distances for each DFA. (PDF)
