# Supplementary material for: The Taxonomic and Phylogenetic Affinities of Bunopithecus sericus, a Fossil Hylobatid from the Pleistocene of China
Source: PLoS One. 2015 Jul 8;10(7):e0131206. doi: 10.1371/journal.pone.0131206 (PMC4495929; doi:10.1371/journal.pone.0131206)
Supplement: S1 Table — (PDF) [file pone.0131206.s001.pdf]

**S1 Table. Descriptive statistics for $M_2$ size and shape variables in *B. sericus* and extant hylobatid genera.**

| Features* | *B. sericus* | *Hoolock* | | | | *Hylobates* | | | | *Nomascus* | | | | *Symphalangus* | | | |
|---|---|---|---|---|---|---|---|---|---|---|---|---|---|---|---|---|---|
| | | *n* | Mean | SD | Range | *n* | Mean | SD | Range | *n* | Mean | SD | Range | *n* | Mean | SD | Range |
| MDLENGTH | 7.47 | 15 | 7.55 | 0.55 | 6.06-8.31 | 91 | 6.46 | 0.54 | 5.07-8.68 | 17 | 6.98 | 0.37 | 6.41-7.58 | 26 | 8.81 | 0.63 | 7.42-9.73 |
| BLMES | 3.55 | 19 | 4.70 | 1.18 | 3.01-6.35 | 89 | 4.40 | 1.06 | 2.44-7.32 | 17 | 5.56 | 0.28 | 4.97-5.97 | 25 | 5.56 | 1.04 | 3.98-7.17 |
| BLDIS | 4.68 | 19 | 5.16 | 1.07 | 3.56-6.64 | 88 | 4.64 | 0.96 | 2.58-7.63 | 17 | 5.91 | 0.38 | 5.10-6.50 | 24 | 6.33 | 1.02 | 4.68-7.88 |
| ANBCUSP | 102.33 | 15 | 102.01 | 6.85 | 91.41-112.42 | 79 | 95.70 | 6.09 | 81.94-110.41 | 17 | 94.62 | 4.69 | 80.65-101.49 | 25 | 97.68 | 6.51 | 86.33-109.52 |
| ANLCUSP | 76.87 | 15 | 77.89 | 8.26 | 67.65-93.06 | 80 | 82.86 | 8.33 | 33.84-98.67 | 17 | 85.82 | 3.65 | 80.44-95.31 | 25 | 82.01 | 6.35 | 70.37-91.27 |
| ANHYCLD | 36.37 | 19 | 39.46 | 4.40 | 30.54-47.93 | 85 | 37.58 | 5.88 | 23.16-52.50 | 17 | 41.75 | 4.83 | 32.99-51.34 | 24 | 41.86 | 7.07 | 28.62-51.70 |
| ABSAPROTO | 9.56 | 13 | 9.88 | 1.52 | 7.08-12.95 | 90 | 7.16 | 1.22 | 3.88-10.74 | 17 | 7.86 | 1.20 | 5.16-10.02 | 28 | 11.14 | 1.60 | 8.24-15.01 |
| ABSAHYPCD | 7.52 | 13 | 8.49 | 1.88 | 3.87-10.61 | 90 | 5.96 | 0.91 | 3.84-8.83 | 17 | 6.98 | 1.24 | 4.68-9.11 | 28 | 11.38 | 1.78 | 8.77-15.23 |
| ABSAHYPCLD | 5.83 | 13 | 5.54 | 1.42 | 2.77-7.86 | 89 | 4.06 | 1.05 | 1.48-8.87 | 17 | 5.25 | 1.10 | 3.58-7.66 | 28 | 8.28 | 1.63 | 5.10-10.76 |
| ABSAMETA | 9.57 | 13 | 9.42 | 1.46 | 6.18-10.86 | 90 | 7.94 | 1.32 | 4.76-11.61 | 17 | 8.74 | 1.23 | 6.26-10.99 | 28 | 13.15 | 2.00 | 8.74-16.95 |
| ABSAENTO | 7.00 | 13 | 6.97 | 1.15 | 5.14-8.71 | 90 | 4.91 | 1.09 | 2.54-8.50 | 17 | 5.92 | 1.30 | 4.35-8.55 | 28 | 8.03 | 2.23 | 3.70-14.63 |
| OCCLAREA | 39.79 | 19 | 42.19 | 5.97 | 25.88-52.42 | 100 | 30.21 | 4.03 | 18.8-40.01 | 17 | 34.64 | 3.79 | 28.32-39.97 | 28 | 51.81 | 6.23 | 37.76-59.53 |
| ABSATRIGD | 20.58 | 15 | 21.17 | 3.29 | 12.62-26.42 | 91 | 14.94 | 2.10 | 9.91-20.25 | 17 | 18.14 | 2.47 | 14.20-23.74 | 28 | 27.30 | 3.72 | 19.98-32.89 |
| ABSATALD | 19.51 | 15 | 19.34 | 2.44 | 13.27-23.49 | 91 | 15.12 | 2.24 | 8.71-21.07 | 17 | 16.47 | 1.70 | 13.58-19.65 | 28 | 24.47 | 3.09 | 17.99-29.85 |
| RELAPROTO | 0.24 | 13 | 0.25 | 0.02 | 0.22-0.29 | 90 | 0.24 | 0.02 | 0.20-0.32 | 17 | 0.23 | 0.03 | 0.17-0.28 | 28 | 0.22 | 0.02 | 0.18-0.26 |
| RELAHYPCD | 0.19 | 13 | 0.21 | 0.03 | 0.15-0.26 | 90 | 0.20 | 0.02 | 0.15-0.28 | 17 | 0.20 | 0.03 | 0.15-0.25 | 28 | 0.22 | 0.03 | 0.18-0.29 |
| RELAHYPCLD | 0.15 | 13 | 0.14 | 0.02 | 0.10-0.17 | 89 | 0.13 | 0.03 | 0.06-0.24 | 17 | 0.15 | 0.03 | 0.11-0.22 | 28 | 0.16 | 0.02 | 0.11-0.20 |
| RELAMETA | 0.24 | 13 | 0.23 | 0.02 | 0.21-0.26 | 90 | 0.26 | 0.03 | 0.16-0.33 | 17 | 0.25 | 0.03 | 0.18-0.32 | 28 | 0.25 | 0.03 | 0.15-0.30 |
| RELAENTO | 0.18 | 13 | 0.18 | 0.03 | 0.12-0.23 | 90 | 0.16 | 0.03 | 0.08-0.26 | 17 | 0.17 | 0.03 | 0.13-0.22 | 28 | 0.15 | 0.03 | 0.09-0.25 |
| RELATRIGD | 0.52 | 15 | 0.52 | 0.02 | 0.48-0.57 | 91 | 0.50 | 0.03 | 0.44-0.58 | 17 | 0.52 | 0.03 | 0.48-0.59 | 28 | 0.53 | 0.02 | 0.47-0.57 |
| RELATALD | 0.49 | 15 | 0.48 | 0.03 | 0.44-0.52 | 91 | 0.50 | 0.03 | 0.43-0.57 | 17 | 0.48 | 0.03 | 0.42-0.53 | 28 | 0.47 | 0.02 | 0.44-0.52 |

*Linear and area measurements in mm and $mm^2$, respectively; angle measurements in radians
