# Supplementary material for: The Taxonomic and Phylogenetic Affinities of Bunopithecus sericus, a Fossil Hylobatid from the Pleistocene of China
Source: PLoS One. 2015 Jul 8;10(7):e0131206. doi: 10.1371/journal.pone.0131206 (PMC4495929; doi:10.1371/journal.pone.0131206)
Supplement: S2 Table — (PDF) [file pone.0131206.s002.pdf]

S2 Table. Descriptive statistics for M_3 size and shape variables in *B. sericus* and extant hylobatid genera.

Features*	*B. sericus*	*Hoolock*				*Hylobates*				*Nomascus*				*Symphalangus*			
		n	Mean	SD	Range	*n*	Mean	SD	Range	*n*	Mean	SD	Range	*n*	Mean	SD	Range
MDLENGTH	7.39	12	7.34	0.35	6.57-7.91	67	6.18	0.57	4.62-7.32	17	6.53	0.46	5.89-7.39	21	8.64	0.65	7.28-10.23
BLMES	3.79	13	5.03	0.91	3.50-6.24	67	4.57	0.85	2.77-6.07	17	5.56	0.38	4.60-6.00	22	5.67	1.02	3.97-7.28
BLDIS	4.91	13	5.24	1.07	3.49-6.41	68	4.52	0.90	2.90-6.99	17	5.42	0.56	4.42-6.26	22	6.07	1.05	4.05-7.70
ANBCUSP	104.74	12	95.41	7.74	80.80-107.79	61	91.86	6.68	77.24-111.70	17	90.85	5.84	83.96-107.70	20	91.79	7.28	82.20-105.38
ANLCUSP	75.96	12	84.52	7.95	74.17-100.29	61	88.26	6.55	68.50-100.92	17	89.35	5.56	73.60-96.48	20	87.97	7.24	73.74-99.67
ANHYCLD	40.46	12	38.12	7.88	24.05-46.51	62	37.87	7.49	18.44-51.78	16	35.00	8.33	20.66-49.21	21	43.19	7.77	30.46-60.69
ABSAPROTO	9.03	11	9.84	1.84	6.80-12.84	66	7.08	1.22	3.85-10.55	17	8.01	1.28	5.94-10.05	21	10.94	1.70	7.68-13.65
ABSAHYPCD	9.13	11	7.55	1.30	5.10-9.41	66	5.27	1.04	2.52-7.52	17	6.39	1.44	4.19-9.60	21	10.02	2.04	6.48-14.54
ABSAHYPCLD	7.33	11	4.63	0.97	2.25-5.77	60	3.56	1.10	1.08-7.04	16	4.16	1.71	1.42-6.64	21	8.34	2.30	2.67-12.40
ABSAMETA	11.43	11	9.21	1.21	7.35-11.72	66	7.31	1.42	3.72-10.53	17	8.18	1.16	6.73-10.25	21	12.91	2.17	8.82-16.62
ABSAENTO	6.36	11	7.11	1.12	5.97-9.64	66	4.44	1.19	2.29-7.68	17	4.75	1.32	2.00-7.11	21	7.71	3.58	3-15.02
OCCLAREA	42.92	13	39.09	3.47	30.32-44.23	73	27.68	4.03	15.98-35.16	17	31.58	4.35	25.55-39.41	22	49.76	7.17	34.58-60.18
ABSATRIGD	23.18	12	19.34	2.08	16.09-23.46	69	12.98	2.28	6.28-16.97	17	15.11	3.23	10.68-22.57	21	25.93	4.64	18.22-34.17
ABSATALD	20.14	12	19.18	2.80	13.53-23.12	69	14.56	2.24	9.13-19.81	17	16.47	1.86	13.28-19.59	21	24.06	3.39	16.50-29.44
RELAPROTO	0.23	10	0.24	0.05	0.17-0.35	63	0.24	0.03	0.17-0.35	17	0.23	0.03	0.17-0.29	19	0.22	0.03	0.17-0.29
RELAHYPCD	0.23	10	0.18	0.03	0.14-0.23	63	0.18	0.03	0.10-0.26	17	0.18	0.03	0.13-0.25	19	0.20	0.04	0.14-0.27
RELAHYPCLD	0.18	10	0.11	0.02	0.06-0.13	58	0.12	0.03	0.04-0.25	16	0.12	0.05	0.04-0.20	19	0.16	0.04	0.06-0.26
RELAMETA	0.29	10	0.22	0.03	0.17-0.27	63	0.24	0.04	0.15-0.31	17	0.24	0.04	0.19-0.32	19	0.25	0.03	0.19-0.31
RELAENTO	0.16	10	0.18	0.03	0.14-0.23	63	0.15	0.04	0.08-0.27	17	0.14	0.03	0.07-0.18	19	0.15	0.06	0.07-0.26
RELATRIGD	0.58	11	0.47	0.04	0.42-0.56	65	0.43	0.06	0.32-0.57	17	0.43	0.07	0.32-0.56	19	0.51	0.05	0.41-0.64
RELATALD	0.51	11	0.46	0.08	0.38-0.63	65	0.49	0.05	0.39-0.61	17	0.48	0.05	0.36-0.58	19	0.48	0.06	0.39-0.57

*Linear and area measurements in mm and mm^2, respectively; angle measurements in radians
