# Supplementary material for: The Taxonomic and Phylogenetic Affinities of Bunopithecus sericus, a Fossil Hylobatid from the Pleistocene of China
Source: PLoS One. 2015 Jul 8;10(7):e0131206. doi: 10.1371/journal.pone.0131206 (PMC4495929; doi:10.1371/journal.pone.0131206)
Supplement: S3 Table — Left: Mann-Whitney U test values; Right: Bonferroni adjusted p-values with significant values bolded. (PDF) [file pone.0131206.s003.pdf]

**S3 Table. Extant hylobatid variance analysis.** Left: Mann-Whitney *U* test values; Right: Bonferroni adjusted *p*-values with significant values bolded.

Focus variable: $M_2$ MDLENGTH

| | *Hoolock* | *Hylobates* | *Nomascus* | *Symphalangus* |
|---|---|---|---|---|
| *Hoolock* | - | | | |
| *Hylobates* | 110 | - | | |
| *Nomascus* | 39.5 | 306 | - | |
| *Symphalangus* | 28.5 | 10 | 2 | - |

| | *Hoolock* | *Hylobates* | *Nomascus* | *Symphalangus* |
|---|---|---|---|---|
| *Hoolock* | - | | | |
| *Hylobates* | < **0.001** | - | | |
| *Nomascus* | < **0.01** | < **0.001** | - | |
| *Symphalangus* | < **0.001** | < **0.001** | < **0.001** | - |

Focus variable: $M_2$ BLMES

| | *Hoolock* | *Hylobates* | *Nomascus* | *Symphalangus* |
|---|---|---|---|---|
| *Hoolock* | - | | | |
| *Hylobates* | 678 | - | | |
| *Nomascus* | 109.5 | 248.5 | - | |
| *Symphalangus* | 122.5 | 476.5 | 190 | - |

| | *Hoolock* | *Hylobates* | *Nomascus* | *Symphalangus* |
|---|---|---|---|---|
| *Hoolock* | - | | | |
| *Hylobates* | 1 | - | | |
| *Nomascus* | 0.6155 | < **0.001** | - | |
| *Symphalangus* | < **0.05** | < **0.001** | 1 | - |

Focus variable: $M_2$ BLDIS

| | *Hoolock* | *Hylobates* | *Nomascus* | *Symphalangus* |
|---|---|---|---|---|
| *Hoolock* | - | | | |
| *Hylobates* | 585 | - | | |
| *Nomascus* | 108.5 | 146.5 | - | |
| *Symphalangus* | 85.5 | 301 | 154.5 | - |

| | *Hoolock* | *Hylobates* | *Nomascus* | *Symphalangus* |
|---|---|---|---|---|
| *Hoolock* | - | | | |
| *Hylobates* | 0.2468 | - | | |
| *Nomascus* | 0.5769 | < **0.001** | - | |
| *Symphalangus* | < **0.01** | < **0.001** | 1 | - |

Focus variable: $M_2$ ANBCUSP

| | *Hoolock* | *Hylobates* | *Nomascus* | *Symphalangus* |
|---|---|---|---|---|
| *Hoolock* | - | | | |
| *Hylobates* | 305 | - | | |
| *Nomascus* | 56.5 | 631.5 | - | |
| *Symphalangus* | 119 | 816.5 | 179 | - |

| | *Hoolock* | *Hylobates* | *Nomascus* | *Symphalangus* |
|---|---|---|---|---|
| *Hoolock* | - | | | |
| *Hylobates* | **< 0.05** | - | | |
| *Nomascus* | **< 0.05** | 1 | - | |
| *Symphalangus* | 0.3448 | 1 | 1 | - |

Focus variable: $M_2$ ANLCUSP

| | *Hoolock* | *Hylobates* | *Nomascus* | *Symphalangus* |
|---|---|---|---|---|
| *Hoolock* | - | | | |
| *Hylobates* | 369 | - | | |
| *Nomascus* | 57 | 520.5 | - | |
| *Symphalangus* | 128 | 903.5 | 146.5 | - |

| | *Hoolock* | *Hylobates* | *Nomascus* | *Symphalangus* |
|---|---|---|---|---|
| *Hoolock* | - | | | |
| *Hylobates* | 0.1119 | - | | |
| *Nomascus* | **< 0.05** | 0.7882 | - | |
| *Symphalangus* | 0.5958 | 1 | 0.5595 | - |

Focus variable: $M_2$ ANHYCLD

| | *Hoolock* | *Hylobates* | *Nomascus* | *Symphalangus* |
|---|---|---|---|---|
| *Hoolock* | - | | | |
| *Hylobates* | 637.5 | - | | |
| *Nomascus* | 113 | 423 | - | |
| *Symphalangus* | 174 | 647 | 197.5 | - |

| | *Hoolock* | *Hylobates* | *Nomascus* | *Symphalangus* |
|---|---|---|---|---|
| *Hoolock* | - | | | |
| *Hylobates* | 0.9234 | - | | |
| *Nomascus* | 0.7696 | **< 0.05** | - | |
| *Symphalangus* | 1 | **< 0.05** | 1 | - |

Focus variable: $M_2$ ABSAPROTO

| | *Hoolock* | *Hylobates* | *Nomascus* | *Symphalangus* |
|---|---|---|---|---|
| *Hoolock* | - | | | |
| *Hylobates* | 95 | - | | |
| *Nomascus* | 30 | 498 | - | |
| *Symphalangus* | 98 | 61.5 | 18.5 | - |

| | *Hoolock* | *Hylobates* | *Nomascus* | *Symphalangus* |
|---|---|---|---|---|
| *Hoolock* | - | | | |
| *Hylobates* | < **0.001** | - | | |
| *Nomascus* | < **0.01** | 0.1388 | - | |
| *Symphalangus* | 0.1159 | < **0.001** | < **0.001** | - |

Focus variable: $M_2$ ABSAHYPCD

| | *Hoolock* | *Hylobates* | *Nomascus* | *Symphalangus* |
|---|---|---|---|---|
| *Hoolock* | - | | | |
| *Hylobates* | 123 | - | | |
| *Nomascus* | 47.5 | 389.5 | - | |
| *Symphalangus* | 45.5 | 1 | 2 | - |

| | *Hoolock* | *Hylobates* | *Nomascus* | *Symphalangus* |
|---|---|---|---|---|
| *Hoolock* | - | | | |
| *Hylobates* | < **0.001** | - | | |
| *Nomascus* | 0.05338 | < **0.01** | - | |
| *Symphalangus* | < **0.001** | < **0.001** | < **0.001** | - |

Focus variable: $M_2$ ABSAHYPCLD

| | *Hoolock* | *Hylobates* | *Nomascus* | *Symphalangus* |
|---|---|---|---|---|
| *Hoolock* | - | | | |
| *Hylobates* | 210 | - | | |
| *Nomascus* | 91 | 294 | - | |
| *Symphalangus* | 41 | 35 | 35.5 | - |

| | *Hoolock* | *Hylobates* | *Nomascus* | *Symphalangus* |
|---|---|---|---|---|
| *Hoolock* | - | | | |
| *Hylobates* | < **0.01** | - | | |
| *Nomascus* | 1 | < **0.001** | - | |
| *Symphalangus* | < **0.001** | < **0.001** | < **0.001** | - |

Focus variable: $M_2$ ABSAMETA

| | *Hoolock* | *Hylobates* | *Nomascus* | *Symphalangus* |
|---|---|---|---|---|
| *Hoolock* | - | | | |
| *Hylobates* | 246.5 | - | | |
| *Nomascus* | 74 | 505 | - | |
| *Symphalangus* | 27 | 46.5 | 17 | - |

| | *Hoolock* | *Hylobates* | *Nomascus* | *Symphalangus* |
|---|---|---|---|---|
| *Hoolock* | - | | | |
| *Hylobates* | **< 0.01** | - | | |
| *Nomascus* | 0.7906 | 0.162 | - | |
| *Symphalangus* | **< 0.001** | **< 0.001** | **< 0.001** | - |

Focus variable: $M_2$ ABSAENTO

| | *Hoolock* | *Hylobates* | *Nomascus* | *Symphalangus* |
|---|---|---|---|---|
| *Hoolock* | - | | | |
| *Hylobates* | 112 | - | | |
| *Nomascus* | 58 | 420 | - | |
| *Symphalangus* | 119 | 223 | 95 | - |

| | *Hoolock* | *Hylobates* | *Nomascus* | *Symphalangus* |
|---|---|---|---|---|
| *Hoolock* | - | | | |
| *Hylobates* | **< 0.001** | - | | |
| *Nomascus* | 0.1772 | **< 0.05** | - | |
| *Symphalangus* | 0.4796 | **< 0.001** | **< 0.01** | - |

Focus variable: $M_2$ OCCLAREA

| | *Hoolock* | *Hylobates* | *Nomascus* | *Symphalangus* |
|---|---|---|---|---|
| *Hoolock* | - | | | |
| *Hylobates* | 117 | - | | |
| *Nomascus* | 42 | 363 | - | |
| *Symphalangus* | 68 | 4 | 7 | - |

| | *Hoolock* | *Hylobates* | *Nomascus* | *Symphalangus* |
|---|---|---|---|---|
| *Hoolock* | - | | | |
| *Hylobates* | **< 0.001** | - | | |
| *Nomascus* | **< 0.001** | **< 0.01** | - | |
| *Symphalangus* | **< 0.001** | **< 0.001** | **< 0.001** | - |

Focus variable: $M_2$ ABSATRIGD

| | *Hoolock* | *Hylobates* | *Nomascus* | *Symphalangus* |
|---|---|---|---|---|
| *Hoolock* | - | | | |
| *Hylobates* | 150 | - | | |
| *Nomascus* | 39 | 476 | - | |
| *Symphalangus* | 41.5 | 18 | 9 | - |

| | *Hoolock* | *Hylobates* | *Nomascus* | *Symphalangus* |
|---|---|---|---|---|
| *Hoolock* | - | | | |
| *Hylobates* | **< 0.001** | - | | |
| *Nomascus* | **< 0.01** | **< 0.05** | - | |
| *Symphalangus* | **< 0.001** | **< 0.001** | **< 0.001** | - |

Focus variable: $M_2$ ABSATALD

| | *Hoolock* | *Hylobates* | *Nomascus* | *Symphalangus* |
|---|---|---|---|---|
| *Hoolock* | - | | | |
| *Hylobates* | 95 | - | | |
| *Nomascus* | 49 | 237.5 | - | |
| *Symphalangus* | 51 | 1 | 12 | - |

| | *Hoolock* | *Hylobates* | *Nomascus* | *Symphalangus* |
|---|---|---|---|---|
| *Hoolock* | - | | | |
| *Hylobates* | **< 0.001** | - | | |
| *Nomascus* | **< 0.05** | **< 0.001** | - | |
| *Symphalangus* | **< 0.001** | **< 0.001** | **< 0.001** | - |

Focus variable: $M_2$ RELAPROTO

| | *Hoolock* | *Hylobates* | *Nomascus* | *Symphalangus* |
|---|---|---|---|---|
| *Hoolock* | - | | | |
| *Hylobates* | 457.5 | - | | |
| *Nomascus* | 59 | 554 | - | |
| *Symphalangus* | 46 | 541 | 167 | - |

| | *Hoolock* | *Hylobates* | *Nomascus* | *Symphalangus* |
|---|---|---|---|---|
| *Hoolock* | - | | | |
| *Hylobates* | 1 | - | | |
| *Nomascus* | 0.1968 | 0.437 | - | |
| *Symphalangus* | **< 0.001** | **< 0.001** | 0.5931 | - |

Focus variable: $M_2$ RELAHYPCD

| | *Hoolock* | *Hylobates* | *Nomascus* | *Symphalangus* |
|---|---|---|---|---|
| *Hoolock* | - | | | |
| *Hylobates* | 430 | - | | |
| *Nomascus* | 93 | 705.5 | - | |
| *Symphalangus* | 139 | 689 | 150 | - |

| | *Hoolock* | *Hylobates* | *Nomascus* | *Symphalangus* |
|---|---|---|---|---|
| *Hoolock* | - | | | |
| *Hylobates* | 0.7497 | - | | |
| *Nomascus* | 1 | 1 | - | |
| *Symphalangus* | 1 | < **0.01** | 0.2431 | - |

Focus variable: $M_2$ RELAHYPCLD

| | *Hoolock* | *Hylobates* | *Nomascus* | *Symphalangus* |
|---|---|---|---|---|
| *Hoolock* | - | | | |
| *Hylobates* | 521 | - | | |
| *Nomascus* | 65 | 478 | - | |
| *Symphalangus* | 80 | 530.5 | 168 | - |

| | *Hoolock* | *Hylobates* | *Nomascus* | *Symphalangus* |
|---|---|---|---|---|
| *Hoolock* | - | | | |
| *Hylobates* | 1 | - | | |
| *Nomascus* | 0.3579 | 0.1001 | - | |
| *Symphalangus* | < **0.05** | < **0.001** | 0.6224 | - |

Focus variable: $M_2$ RELAMETA

| | *Hoolock* | *Hylobates* | *Nomascus* | *Symphalangus* |
|---|---|---|---|---|
| *Hoolock* | - | | | |
| *Hylobates* | 176 | - | | |
| *Nomascus* | 67 | 590 | - | |
| *Symphalangus* | 75 | 953 | 224 | - |

| | *Hoolock* | *Hylobates* | *Nomascus* | *Symphalangus* |
|---|---|---|---|---|
| *Hoolock* | - | | | |
| *Hylobates* | < **0.001** | - | | |
| *Nomascus* | 0.4315 | 0.822 | - | |
| *Symphalangus* | < **0.05** | 0.3151 | 1 | - |

Focus variable: $M_2$ RELAENTO

| | *Hoolock* | *Hylobates* | *Nomascus* | *Symphalangus* |
|---|---|---|---|---|
| *Hoolock* | - | | | |
| *Hylobates* | 453 | - | | |
| *Nomascus* | 91 | 686 | - | |
| *Symphalangus* | 99 | 964 | 153 | - |

| | *Hoolock* | *Hylobates* | *Nomascus* | *Symphalangus* |
|---|---|---|---|---|
| *Hoolock* | - | | | |
| *Hylobates* | 1 | - | | |
| *Nomascus* | 1 | 1 | - | |
| *Symphalangus* | 0.1249 | 0.3695 | 0.2875 | - |

Focus variable: $M_2$ RELATRIGD

| | *Hoolock* | *Hylobates* | *Nomascus* | *Symphalangus* |
|---|---|---|---|---|
| *Hoolock* | - | | | |
| *Hylobates* | 359 | - | | |
| *Nomascus* | 124 | 404 | - | |
| *Symphalangus* | 190 | 544 | 210 | - |

| | *Hoolock* | *Hylobates* | *Nomascus* | *Symphalangus* |
|---|---|---|---|---|
| *Hoolock* | - | | | |
| *Hylobates* | **< 0.05** | - | | |
| *Nomascus* | 1 | **< 0.05** | - | |
| *Symphalangus* | 1 | **< 0.001** | 1 | - |

Focus variable: $M_2$ RELATALD

| | *Hoolock* | *Hylobates* | *Nomascus* | *Symphalangus* |
|---|---|---|---|---|
| *Hoolock* | - | | | |
| *Hylobates* | 387 | - | | |
| *Nomascus* | 127 | 417 | - | |
| *Symphalangus* | 184 | 570 | 195 | - |

| | *Hoolock* | *Hylobates* | *Nomascus* | *Symphalangus* |
|---|---|---|---|---|
| *Hoolock* | - | | | |
| *Hylobates* | **< 0.05** | - | | |
| *Nomascus* | 1 | **< 0.05** | - | |
| *Symphalangus* | 1 | **< 0.001** | 1 | - |

Focus variable: $M_3$ MDLENGTH

| | *Hoolock* | *Hylobates* | *Nomascus* | *Symphalangus* |
|---|---|---|---|---|
| *Hoolock* | - | | | |
| *Hylobates* | 23.5 | - | | |
| *Nomascus* | 17 | 378 | - | |
| *Symphalangus* | 10.5 | 1 | 2 | - |

| | *Hoolock* | *Hylobates* | *Nomascus* | *Symphalangus* |
|---|---|---|---|---|
| *Hoolock* | - | | | |
| *Hylobates* | < **0.001** | - | | |
| *Nomascus* | < **0.01** | 0.2007 | - | |
| *Symphalangus* | < **0.001** | < **0.001** | < **0.001** | - |

Focus variable: $M_3$ BLMES

| | *Hoolock* | *Hylobates* | *Nomascus* | *Symphalangus* |
|---|---|---|---|---|
| *Hoolock* | - | | | |
| *Hylobates* | 283.5 | - | | |
| *Nomascus* | 76.5 | 147.5 | - | |
| *Symphalangus* | 81 | 323.5 | 148 | - |

| | *Hoolock* | *Hylobates* | *Nomascus* | *Symphalangus* |
|---|---|---|---|---|
| *Hoolock* | - | | | |
| *Hylobates* | 0.2889 | - | | |
| *Nomascus* | 0.9646 | < **0.001** | - | |
| *Symphalangus* | 0.2144 | < **0.001** | 1 | - |

Focus variable: $M_3$ BLDIS

| | *Hoolock* | *Hylobates* | *Nomascus* | *Symphalangus* |
|---|---|---|---|---|
| *Hoolock* | - | | | |
| *Hylobates* | 250 | - | | |
| *Nomascus* | 109 | 231.5 | - | |
| *Symphalangus* | 95.5 | 198.5 | 116 | - |

| | *Hoolock* | *Hylobates* | *Nomascus* | *Symphalangus* |
|---|---|---|---|---|
| *Hoolock* | - | | | |
| *Hylobates* | 0.08241 | - | | |
| *Nomascus* | 1 | < **0.001** | - | |
| *Symphalangus* | 0.6513 | < **0.001** | 0.2751 | - |

Focus variable: $M_3$ ANBCUSP

| | *Hoolock* | *Hylobates* | *Nomascus* | *Symphalangus* |
|---|---|---|---|---|
| *Hoolock* | - | | | |
| *Hylobates* | 264.5 | - | | |
| *Nomascus* | 61 | 454.5 | - | |
| *Symphalangus* | 84 | 578 | 169 | - |

| | *Hoolock* | *Hylobates* | *Nomascus* | *Symphalangus* |
|---|---|---|---|---|
| *Hoolock* | - | | | |
| *Hylobates* | 0.7966 | - | | |
| *Nomascus* | 0.4375 | 1 | - | |
| *Symphalangus* | 1 | 1 | 1 | - |

Focus variable: $M_3$ ANLCUSP

| | *Hoolock* | *Hylobates* | *Nomascus* | *Symphalangus* |
|---|---|---|---|---|
| *Hoolock* | - | | | |
| *Hylobates* | 248 | - | | |
| *Nomascus* | 63 | 475 | - | |
| *Symphalangus* | 89 | 600.5 | 149 | - |

| | *Hoolock* | *Hylobates* | *Nomascus* | *Symphalangus* |
|---|---|---|---|---|
| *Hoolock* | - | | | |
| *Hylobates* | 0.4819 | - | | |
| *Nomascus* | 0.5291 | 1 | - | |
| *Symphalangus* | 1 | 1 | 1 | - |

Focus variable: $M_3$ ANHYCLD

| | *Hoolock* | *Hylobates* | *Nomascus* | *Symphalangus* |
|---|---|---|---|---|
| *Hoolock* | - | | | |
| *Hylobates* | 344.5 | - | | |
| *Nomascus* | 74 | 403.5 | - | |
| *Symphalangus* | 89 | 441 | 81 | - |

| | *Hoolock* | *Hylobates* | *Nomascus* | *Symphalangus* |
|---|---|---|---|---|
| *Hoolock* | - | | | |
| *Hylobates* | 1 | - | | |
| *Nomascus* | 1 | 0.7317 | - | |
| *Symphalangus* | 1 | 0.1383 | 0.02642 | - |

Focus variable: $M_3$ ABSAPROTO

| | *Hoolock* | *Hylobates* | *Nomascus* | *Symphalangus* |
|---|---|---|---|---|
| *Hoolock* | - | | | |
| *Hylobates* | 86 | - | | |
| *Nomascus* | 36 | 332 | - | |
| *Symphalangus* | 75 | 59 | 29.5 | - |

| | *Hoolock* | *Hylobates* | *Nomascus* | *Symphalangus* |
|---|---|---|---|---|
| *Hoolock* | - | | | |
| *Hylobates* | **< 0.001** | - | | |
| *Nomascus* | **< 0.05** | 0.05952 | - | |
| *Symphalangus* | 0.675 | **< 0.001** | **< 0.001** | - |

Focus variable: $M_3$ ABSAHYPCD

| | *Hoolock* | *Hylobates* | *Nomascus* | *Symphalangus* |
|---|---|---|---|---|
| *Hoolock* | - | | | |
| *Hylobates* | 65.5 | - | | |
| *Nomascus* | 49.5 | 312.5 | - | |
| *Symphalangus* | 34 | 10.5 | 24 | - |

| | *Hoolock* | *Hylobates* | *Nomascus* | *Symphalangus* |
|---|---|---|---|---|
| *Hoolock* | - | | | |
| *Hylobates* | **< 0.001** | - | | |
| *Nomascus* | 0.2442 | **< 0.05** | - | |
| *Symphalangus* | **< 0.01** | **< 0.001** | **< 0.001** | - |

Focus variable: $M_3$ ABSAHYPCLD

| | *Hoolock* | *Hylobates* | *Nomascus* | *Symphalangus* |
|---|---|---|---|---|
| *Hoolock* | - | | | |
| *Hylobates* | 150 | - | | |
| *Nomascus* | 73.5 | 357.5 | - | |
| *Symphalangus* | 10 | 52 | 17 | - |

| | *Hoolock* | *Hylobates* | *Nomascus* | *Symphalangus* |
|---|---|---|---|---|
| *Hoolock* | - | | | |
| *Hylobates* | **< 0.05** | - | | |
| *Nomascus* | 1 | 0.7204 | - | |
| *Symphalangus* | **< 0.001** | **< 0.001** | **< 0.001** | - |

Focus variable: $M_3$ ABSAMETA

| | *Hoolock* | *Hylobates* | *Nomascus* | *Symphalangus* |
|---|---|---|---|---|
| *Hoolock* | - | | | |
| *Hylobates* | 104.5 | - | | |
| *Nomascus* | 48.5 | 379 | - | |
| *Symphalangus* | 17 | 19 | 12 | - |

| | *Hoolock* | *Hylobates* | *Nomascus* | *Symphalangus* |
|---|---|---|---|---|
| *Hoolock* | - | | | |
| *Hylobates* | **< 0.01** | - | | |
| *Nomascus* | 0.2178 | 0.2433 | - | |
| *Symphalangus* | **< 0.001** | **< 0.001** | **< 0.001** | - |

Focus variable: $M_3$ ABSAENTO

| | *Hoolock* | *Hylobates* | *Nomascus* | *Symphalangus* |
|---|---|---|---|---|
| *Hoolock* | - | | | |
| *Hylobates* | 30 | - | | |
| *Nomascus* | 10 | 456.5 | - | |
| *Symphalangus* | 114 | 312.5 | 102.5 | - |

| | *Hoolock* | *Hylobates* | *Nomascus* | *Symphalangus* |
|---|---|---|---|---|
| *Hoolock* | - | | | |
| *Hylobates* | **< 0.001** | - | | |
| *Nomascus* | **< 0.001** | 1 | - | |
| *Symphalangus* | 1 | **< 0.001** | 0.1599 | - |

Focus variable: $M_3$ OCCLAREA

| | *Hoolock* | *Hylobates* | *Nomascus* | *Symphalangus* |
|---|---|---|---|---|
| *Hoolock* | - | | | |
| *Hylobates* | 20 | - | | |
| *Nomascus* | 20 | 359 | - | |
| *Symphalangus* | 29.5 | 1 | 6 | - |

| | *Hoolock* | *Hylobates* | *Nomascus* | *Symphalangus* |
|---|---|---|---|---|
| *Hoolock* | - | | | |
| *Hylobates* | **< 0.001** | - | | |
| *Nomascus* | **< 0.001** | **< 0.05** | - | |
| *Symphalangus* | **< 0.001** | **< 0.001** | **< 0.001** | - |

Focus variable: $M_3$ ABSATRIGD

| | *Hoolock* | *Hylobates* | *Nomascus* | *Symphalangus* |
|---|---|---|---|---|
| *Hoolock* | - | | | |
| *Hylobates* | 83.5 | - | | |
| *Nomascus* | 42.5 | 306 | - | |
| *Symphalangus* | 33 | 17 | 15 | - |

| | *Hoolock* | *Hylobates* | *Nomascus* | *Symphalangus* |
|---|---|---|---|---|
| *Hoolock* | - | | | |
| *Hylobates* | **< 0.001** | - | | |
| *Nomascus* | 0.05387 | **< 0.05** | - | |
| *Symphalangus* | **< 0.01** | **< 0.001** | **< 0.001** | - |

Focus variable: $M_3$ ABSATALD

| | *Hoolock* | *Hylobates* | *Nomascus* | *Symphalangus* |
|---|---|---|---|---|
| *Hoolock* | - | | | |
| *Hylobates* | 7 | - | | |
| *Nomascus* | 27 | 373.5 | - | |
| *Symphalangus* | 29 | 0 | 11 | - |

| | *Hoolock* | *Hylobates* | *Nomascus* | *Symphalangus* |
|---|---|---|---|---|
| *Hoolock* | - | | | |
| *Hylobates* | **< 0.001** | - | | |
| *Nomascus* | **< 0.01** | 0.1272 | - | |
| *Symphalangus* | **< 0.01** | **< 0.001** | **< 0.001** | - |

Focus variable: $M_3$ RELAPROTO

| | *Hoolock* | *Hylobates* | *Nomascus* | *Symphalangus* |
|---|---|---|---|---|
| *Hoolock* | - | | | |
| *Hylobates* | 306 | - | | |
| *Nomascus* | 75 | 482 | - | |
| *Symphalangus* | 67 | 405 | 120 | - |

| | *Hoolock* | *Hylobates* | *Nomascus* | *Symphalangus* |
|---|---|---|---|---|
| *Hoolock* | - | | | |
| *Hylobates* | 1 | - | | |
| *Nomascus* | 1 | 1 | - | |
| *Symphalangus* | 1 | 0.2035 | 1 | - |

Focus variable: $M_3$ RELAHYPCD

| | *Hoolock* | *Hylobates* | *Nomascus* | *Symphalangus* |
|---|---|---|---|---|
| *Hoolock* | - | | | |
| *Hylobates* | 254 | - | | |
| *Nomascus* | 84 | 442.5 | - | |
| *Symphalangus* | 76 | 388 | 125 | - |

| | *Hoolock* | *Hylobates* | *Nomascus* | *Symphalangus* |
|---|---|---|---|---|
| *Hoolock* | - | | | |
| *Hylobates* | 1 | - | | |
| *Nomascus* | 1 | 1 | - | |
| *Symphalangus* | 1 | 0.126 | 1 | - |

Focus variable: $M_3$ RELAHYPCLD

| | *Hoolock* | *Hylobates* | *Nomascus* | *Symphalangus* |
|---|---|---|---|---|
| *Hoolock* | - | | | |
| *Hylobates* | 255 | - | | |
| *Nomascus* | 63 | 416 | - | |
| *Symphalangus* | 17 | 189 | 76 | - |

| | *Hoolock* | *Hylobates* | *Nomascus* | *Symphalangus* |
|---|---|---|---|---|
| *Hoolock* | - | | | |
| *Hylobates* | 1 | - | | |
| *Nomascus* | 1 | 1 | - | |
| *Symphalangus* | < **0.01** | < **0.001** | 0.0745 | - |

Focus variable: $M_3$ RELAMETA

| | *Hoolock* | *Hylobates* | *Nomascus* | *Symphalangus* |
|---|---|---|---|---|
| *Hoolock* | - | | | |
| *Hylobates* | 183 | - | | |
| *Nomascus* | 59 | 438 | - | |
| *Symphalangus* | 45 | 513 | 110 | - |

| | *Hoolock* | *Hylobates* | *Nomascus* | *Symphalangus* |
|---|---|---|---|---|
| *Hoolock* | - | | | |
| *Hylobates* | 0.2093 | - | | |
| *Nomascus* | 1 | 1 | - | |
| *Symphalangus* | 0.1388 | 1 | 0.6365 | - |

Focus variable: $M_3$ RELAENTO

| | *Hoolock* | *Hylobates* | *Nomascus* | *Symphalangus* |
|---|---|---|---|---|
| *Hoolock* | - | | | |
| *Hylobates* | 166 | - | | |
| *Nomascus* | 28 | 425 | - | |
| *Symphalangus* | 75 | 585 | 137 | - |

| | *Hoolock* | *Hylobates* | *Nomascus* | *Symphalangus* |
|---|---|---|---|---|
| *Hoolock* | - | | | |
| *Hylobates* | 0.1032 | - | | |
| *Nomascus* | **< 0.05** | 1 | - | |
| *Symphalangus* | 1 | 1 | 1 | - |

Focus variable: $M_3$ RELATRIGD

| | *Hoolock* | *Hylobates* | *Nomascus* | *Symphalangus* |
|---|---|---|---|---|
| *Hoolock* | - | | | |
| *Hylobates* | 233 | - | | |
| *Nomascus* | 66 | 498.5 | - | |
| *Symphalangus* | 77 | 544 | 154 | - |

| | *Hoolock* | *Hylobates* | *Nomascus* | *Symphalangus* |
|---|---|---|---|---|
| *Hoolock* | - | | | |
| *Hylobates* | 0.4029 | - | | |
| *Nomascus* | 1 | 1 | - | |
| *Symphalangus* | 1 | 1 | 1 | - |

Focus variable: $M_3$ RELATALD

| | *Hoolock* | *Hylobates* | *Nomascus* | *Symphalangus* |
|---|---|---|---|---|
| *Hoolock* | - | | | |
| *Hylobates* | 250 | - | | |
| *Nomascus* | 72 | 533 | - | |
| *Symphalangus* | 56 | 223 | 63 | - |

| | *Hoolock* | *Hylobates* | *Nomascus* | *Symphalangus* |
|---|---|---|---|---|
| *Hoolock* | - | | | |
| *Hylobates* | 0.685 | - | | |
| *Nomascus* | 1 | 1 | - | |
| *Symphalangus* | 0.2331 | **< 0.001** | **< 0.05** | - |
