# Supplementary material for: The Taxonomic and Phylogenetic Affinities of Bunopithecus sericus, a Fossil Hylobatid from the Pleistocene of China
Source: PLoS One. 2015 Jul 8;10(7):e0131206. doi: 10.1371/journal.pone.0131206 (PMC4495929; doi:10.1371/journal.pone.0131206)
Supplement: S4 Table — (PDF) [file pone.0131206.s004.pdf]

S4 Table. Average pair-wise Mahalanobis distances for each DFA.

Distances based on absolute variables in M_2 (see also Fig. 3):

	Bunopithecus	*Hoolock*	*Hylobates*	*Nomascus*	*Symphalangus*
Hoolock	1.203213615	1.268151397	-	-	-
Hylobates	1.251276987	1.360384112	1.237795975	-	-
Nomascus	1.303326294	1.305947403	1.248540015	1.190138860	-
Symphalangus	1.494418333	1.574835503	1.567737094	1.507862728	1.608609493

Distances based on absolute variables in M_3 (see also Fig. 4):

	Bunopithecus	*Hoolock*	*Hylobates*	*Nomascus*	*Symphalangus*
Hoolock	1.315880909	1.382754945	-	-	-
Hylobates	1.310389158	1.348998834	1.208561150	-	-
Nomascus	1.361359375	1.337043531	1.238605232	1.235676367	-
Symphalangus	1.528910450	1.612534515	1.599679379	1.577355644	1.720434874

Distances based on absolute variables in $M_{2\text{-}3}$ (see also Fig. 5):

	Bunopithecus	*Hoolock*	*Hylobates*	*Nomascus*	*Symphalangus*
Hoolock	1.396336667	1.348071944	-	-	-
Hylobates	1.400498654	1.361070252	1.305148483	-	-
Nomascus	1.433930625	1.346777958	1.304851618	1.275286792	-
Symphalangus	1.573886667	1.546036543	1.535476158	1.512375451	1.655666471

Distances based on shape variables in $M_{2\text{-}3}$ (see also Fig. 6):

	Bunopithecus	*Hoolock*	*Hylobates*	*Nomascus*	*Symphalangus*
Hoolock	1.298866667	1.287508361	-	-	-
Hylobates	1.413944808	1.366397288	1.405202258	-	-
Nomascus	1.414656875	1.339523958	1.375278225	1.317103217	-
Symphalangus	1.385725556	1.396725796	1.441401381	1.403067344	1.431606876
